# The Use of Extracellular Vesicles in Achilles Tendon Repair: A Systematic Review

**DOI:** 10.3390/biomedicines12050942

**Published:** 2024-04-23

**Authors:** Varun Kasula, Vikram Padala, Nithin Gupta, David Doyle, Kian Bagheri, Albert Anastasio, Samuel Bruce Adams

**Affiliations:** 1 Department of Orthopedic Surgery, Campbell University School of Osteopathic Medicine, Lillington, NC 27546, USA; 2 Department of Orthopedic Surgery, Lewis Katz School of Medicine at Temple University, Philadelphia, PA 19140, USA; 3 Department of Orthopedic Surgery, Central Michigan University College of Medicine, Saginaw, MI 48602, USA; 4 Department of Orthopedic Surgery, Duke University School of Medicine, Durham, NC 27710, USA

**Keywords:** Achilles tendon, extracellular vesicle, exosome, mesenchymal stem cell, wound healing

## Abstract

Achilles tendon (AT) pathologies are common musculoskeletal conditions that can significantly impair function. Despite various traditional treatments, recovery is often slow and may not restore full functionality. The use of extracellular vesicles (EVs) has emerged as a promising therapeutic option due to their role in cell signaling and tissue regeneration. This systematic review aims to consolidate current in vivo animal study findings on the therapeutic effects of EVs on AT injuries. An extensive literature search was conducted using the PubMed, Scopus, and Embase databases for in vivo animal studies examining the effects of EVs on AT pathologies. The extracted variables included but were not limited to the study design, type of EVs used, administration methods, efficacy of treatment, and proposed therapeutic mechanisms. After screening, 18 studies comprising 800 subjects were included. All but one study reported that EVs augmented wound healing processes in the AT. The most proposed mechanisms through which this occurred were gene regulation of the extracellular matrix (ECM), the enhancement of macrophage polarization, and the delivery of therapeutic microRNAs to the injury site. Further research is warranted to not only explore the therapeutic potential of EVs in the context of AT pathologies, but also to establish protocols for their clinical application.

## 1. Introduction

The Achilles tendon (AT), the strongest and thickest tendon in the human body, has attracted considerable interest due to its susceptibility to injury [1,2,3]. This vulnerability is especially noteworthy among physically active individuals, such as athletes and military personnel [4,5]. The most common area for occurrence of an AT rupture is 2–6 cm proximal to the calcaneal insertion, or generally in the middle third of the tendon, which is regarded as a watershed area due to its relative hypovascularity [6]. In younger individuals, AT injuries frequently result from sports participation and manifest abruptly with noticeable symptoms, such as swelling, pain, or difficulty in movement, whereas in older individuals and those with higher body mass indices (BMIs), AT injuries are primarily associated with non-sport activities and delayed detection, commonly resulting in a postponed diagnosis [4,7,8]. In both populations, AT injury features a characteristic cascade, whereby the slow progress of healing and the weakened ability of the AT to bear mechanical stress complicates current treatment schemes [9].

Following AT injury, the majority of individuals can achieve recovery through non-surgical means. However, for those who fail conservative therapy, surgical interventions may be necessary for full recovery [10,11]. Current rehabilitation methods focus on functional braces and early tendon motion and loading, where symptom modification may be achieved through topical, oral, or injected medications, as well as cold therapy, orthotics, and targeted manual techniques [11,12]. Injected corticosteroids may assist with acute tendon pain, but there is a concern about their ability to alter the tendon structure and function, potentially resulting in tearing or rupture. Prior corticosteroid use has been related to drug-induced tendinopathy [13,14,15]. Moreover, the open surgical repair of a ruptured AT has been shown to significantly reduce the re-rupture rate compared with nonoperative treatment. Despite this, surgical treatment is linked to a higher wound complication rate that may be mitigated through the use of minimally invasive surgical techniques [16,17].

Although current treatments exist, once injured, the AT is altered at the cellular and extracellular levels through inflammatory and fibrotic changes, which fundamentally alter the tendon morphology [18,19]. As such, there is currently a focus on the identification of treatments that may assist in the cellular cascades related to AT injury by preserving AT morphology and function. To combat these changes, cellular therapies have been suggested as they have shown efficacy in maintaining tendon health [20,21]. Notably, extracellular vesicles (EV) are of particular promise within AT restoration, where EVs can attenuate the deleterious immune cascades, accelerate tendon angiogenesis, and preserve tendon integrity [22,23,24].

EVs are small, membrane-bound particles released by cells into the extracellular environment, containing a variety of molecular constituents of their cell of origin, including proteins, lipids, RNA, and DNA [25,26,27]. They play a crucial role in cell-to-cell communication and are involved in various physiological and pathological processes. EVs exist in three main forms: exosomes, microvesicles or ectosomes, and apoptotic bodies. Their characterization is primarily based on their size and origin. Exosomes have the smallest diameter at 30 to 150 nm, microvesicles are intermediate in size at a diameter of 100–1000 nm, and apoptotic bodies contain the largest diameter at 500 to 5000 nm [25]. Within cells, exosomes are produced by the endosomal, multivesicular body pathway; ectosomes are created from the budding and fission of the plasma membrane; and apoptotic bodies are produced during apoptosis [26,28]. Through the delivery of their various contents, EVs have been proposed to exert therapeutic influences via immunomodulatory effects, the stimulation of cell proliferation, extracellular matrix remodeling, and through other mechanisms as well [20,22,23,24,25,26].

In regard to their application in treating AT injury, as tenocyte numbers decrease during injury, natural EVs decrease, leading to an inability to support localized healing. This then leads to further detrimental aspects of the inflammatory response and an alteration in the cellular environment [29]. The use of EV-based treatment modalities has gained prominence for its capacity to modify pathologies such as AT disease, altering disease progression through their influence on immunological pathways, cellular microenvironments, and other pertinent components of cellular communication [4,9]. EVs demonstrate low immunogenicity, strong tissue penetration, and the ability to promote tissue regeneration and functional restoration [9,27]. Thus, research has focused on the restoration or supplementation of EVs within AT injury. Due to the relative novelty of applying EVs to AT pathologies, the purpose of this study is to systematically review the current literature to provide a thorough overview of the current state of EVs as potential therapeutic tools for AT, along with the barriers to their clinical integration and future directions.

## 2. Materials and Methods

A systematic review was performed in accordance with the PRISMA guidelines, as outlined with further methodology in the Supplementary Materials (Table S1).

## 3. Results

### 3.1. Search Results and Quality of Included Studies

**Figure 1:**
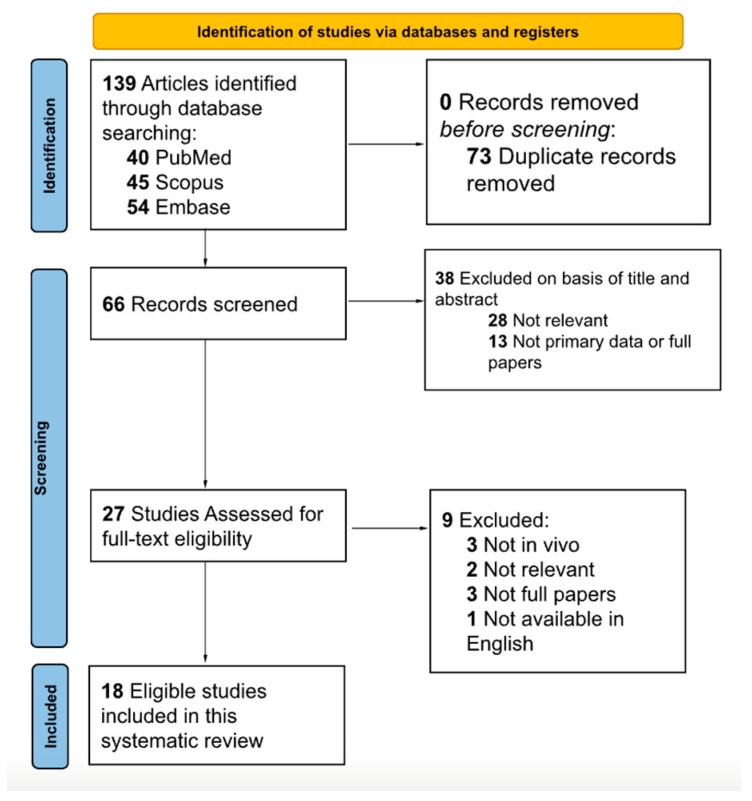
PRISMA diagram showing study selection process.

**Figure 2:**
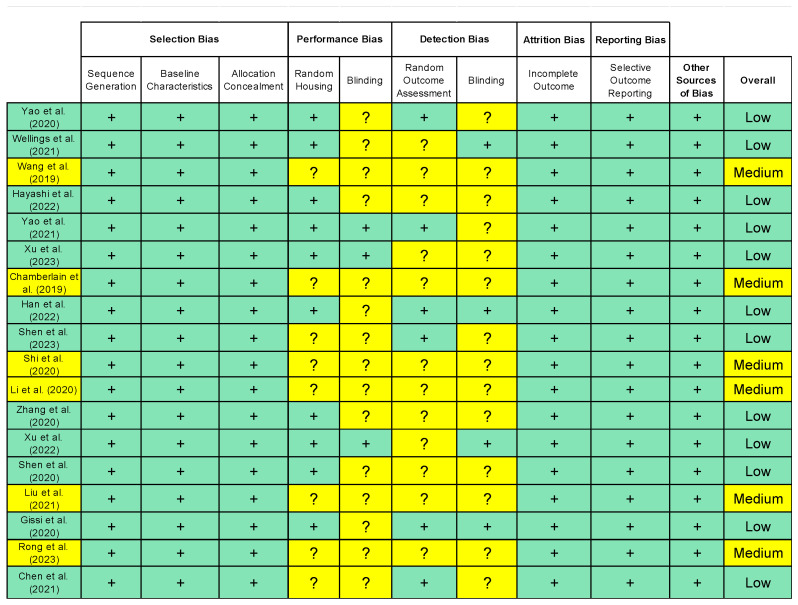
Quality assessment of included studies using SYRCLE risk of bias tool. Green boxes indicate no concerns; yellow boxes indicate some concerns [9,22,23,24,30,31,32,33,34,35,36,37,38,39,40,41,42,43].

A total of 139 studies were extracted from the database search. After duplicates were deleted, 66 studies remained. A title and abstract screen then resulted in 27 remaining studies. While assessing for full-text eligibility, nine more studies were excluded. Ultimately, 18 eligible studies consisting of 800 total subjects were included in this systematic review (Figure 1). The majority of the studies (66.67%) were deemed to have an overall low risk of bias, while the rest of the studies (33.33%) were considered to have a medium risk of bias. The screening strategy and quality assessment are summarized inFigure 1 and Figure 2. Due to the considerable heterogeneity among the included studies in terms of sampling techniques, outcome measures, and study designs, it was deemed not feasible to conduct a pooled meta-analysis without compromising the validity of the results.

### 3.2. Study Characteristics

Of the included studies, 11 are from China and 4 are from the USA, and there is 1 each from Japan, Italy, and Taiwan (Table 1). All 18 included studies were conducted in vivo, with 11 studies using a rat model (*n* = 503), 5 studies using a mouse model (*n* = 196), and 2 studies using a rabbit model (*n* ≥ 81). Regarding the type of EV used, 15 studies only used exosomes, while Xu et al. conducted the only study that compared the effectiveness of both exosomes and microvesicles. Two studies used extracellular vesicles, but the specific type was unspecified.

All but one study used mesenchymal stem cells (MSCs) to isolate the extracellular vesicles. A total of 10 studies used animal-derived MSCs (*n* = 412), while 7 studies used human-derived MSCs (*n* = 343). Amongst the animal-derived MSC studies, the following types of MSCs were isolated: tendon stem cells (TSCs), bone marrow stem cells (BMSCs), and adipose tissue stem cells (ATSCs). Amongst the human-derived MSC studies, the following types of MSCs were isolated: umbilical cord stem cells (UCSCs), BMSCs, and ATSCs. Wellings et al. conducted the one study that used autologously derived platelets to isolate the exosomes (*n* = 45) [32]. These results are summarized in Table 2.

### 3.3. Mechanism of Injury

Across the reviewed studies, a variety of methods were employed to induce injuries to the AT (Table 2). As previously mentioned, the most common area for an AT rupture occurs in the middle third of the tendon due to its relative hypovascularity [6]. In nine studies, the AT transections were induced in this watershed area. There were three studies (*n* = 235 subjects) in which transection was induced in the distal third of the AT, closer to the calcaneal insertion, and three studies (*n* = 142) in which the exact location of injury was unclear. In the remaining three studies (*n* = 108), varying amounts of type 1 collagenase was injected into the AT to enzymatically degrade the collagen fibers, inducing a state of tendinopathy.

### 3.4. Treatment Modalities

For the experimental groups, seven studies only utilized stem cell-derived EVs, while other studies sought to enhance the therapeutic potential of EVs via additional modifications (Table 2). For example, five studies incorporated EVs that were loaded onto scaffolding structures such as type 1 collagen sheets and various hydrogels, and three studies primed EVs with inflammatory or anti-tumorigenic stimuli. Interestingly, Shen et al. and Xu et al. employed a combined approach using hydrogels and priming agents to deliver the EVs [23,24]. Notably, a subset of four studies utilized dual experimental groups in which both groups provided novel information regarding EVs. Hayashi et al. contrasted EVs from early passaged versus senescent MSCs [34]. The senescent EVs showed no difference compared to the control group, suggesting that aging mitigates the therapeutic potential of EVs. Furthermore, Shen et al. and Gissi et al. both opted to use two experimental groups in which the only difference was the concentration of stem cell-derived EVs that were used [23,42]. Further details regarding the study methods and designs of the included studies are included in Table 2.

### 3.5. Mechanism of Delivery

The delivery methods that were adopted to introduce extracellular vesicles to Achilles tendon injuries are summarized in Table 2 and generally followed a specific pattern with a few exceptions. All seven studies in which the experimental group was treated with stem cell-derived EVs without additional modifications used injections to deliver the EVs. Meanwhile, all six studies that loaded the EVs onto a type of scaffold either implanted or topically placed the EV-laden scaffold at the injury site. There were three studies which introduced unique mechanisms of EV delivery to the injury site, all of which included external structures made up of various biomaterials to suspend the EVs in with the intention of promoting better tissue penetration and EV exposure.

### 3.6. Outcome of Treatments for Achilles Tendon Injuries

All studies included in this review found that the administration of EVs improved tendon healing in at least one parameter. The outcomes of all studies are listed in Supplementary Table S2. The parameters that were measured included the amount of collagen growth, organization of fibers, tendon adhesion, tensile strength, and others. Most studies investigated and proposed multiple patterns of different gene/protein expression through which EVs may act to influence tendon healing (Table 3). There were seven studies that determined enhanced macrophage polarization—which consists of either decreased M1 macrophage differentiation, increased M2 macrophage differentiation, or both—was a major factor through which EVs improved healing [22,23,34,36,38,40,43]. Meanwhile, 10 studies found an increase in collagen 1 gene expression, a decrease in collagen 3 expression, or an increase in the ratio of collagen 1 expression to collagen 3 expression [9,24,31,32,37,38,39,40,41,43]. The modulation of anti-inflammatory (IL-10, TGF-β, etc.) and/or proinflammatory (IL-1B, IL-6, TGF-α) molecules after the administration of EVs was observed in seven studies [23,31,36,38,40,41,43]. Furthermore, five studies found that EVs modify the expression of various matrix metalloproteinases (MMP-1, MMP-3, MMP-13, etc.) [32,38,40,41,42], while seven studies isolated EV-based microRNAs that modulated the expression of various repair-promoting and/or anti-fibrotic genes [23,31,34,35,39,40]. Finally, enhancements in biomechanical properties were reported in 10 studies [9,22,24,30,31,32,34,36,39,43], while histological improvements were found in 17 studies [9,23,24,30,31,32,33,34,35,36,37,38,39,40,41,42,43] (Supplementary Figure S2).

## 4. Discussion

Overall, our observations overwhelmingly suggest that EVs can exert therapeutic effects on AT healing with minimal negative outcomes and no significant adverse effects reported. In the context of tendinous tissue repair, it is important to understand the three main stages of healing: inflammation, proliferation, and maturation [44,45]. The initial inflammatory response is characterized by the recruitment of immune cells and cytokines in order to prevent potential infections and clear damaged tissue. The proliferative phase focuses on reinstating the structural integrity of the tendon during which fibroblasts and other cellular entities proliferate and migrate, contributing to granulation tissue formation, tenocyte proliferation, and angiogenesis. The final maturation stage typically begins 3 to 4 weeks post-injury and emphasizes extracellular matrix (ECM) remodeling to improve tensile strength and collagen alignment. From cytokine modulation to macrophage polarization to extracellular matrix regulation, our study aims to clarify many of the nuanced patterns of differential gene and protein expression by which EVs can enhance tissue repair and regeneration across all stages of wound healing in AT pathologies. Additionally, we will explore broader topics such as the impact of microRNAs, innovative strategies for EV delivery to injury sites, and the future of EV-based therapies.

### 4.1. Cytokine Modulation

The initial acute inflammatory response orchestrates a crucial cascade of pro-inflammatory cytokines, serving to contain the spread of harmful agents, clear cellular debris, and lay the foundation for tissue repair [46,47]. While this regulated inflammation is crucial for healing, excessive inflammatory responses can impede tendon repair [48]. Our findings indicate that EVs modulate this response primarily through cytokine modulation, thus facilitating the healing of the AT.

Approximately half of the studies in this review identified cytokine modulation as a major bioactive response that EVs may trigger to enhance AT healing [36]. Shi et al. observed that BMSC-derived exosomes upregulated anti-inflammatory cytokines like IL-10 and TGF-β1 while suppressing pro-inflammatory cytokines IL-1β and IL-6. Concurrently, Zhang et al. and Liu et al. documented a downregulation of proinflammatory agents including COX-2 and IL-8 by EVs [38,41]. Moreover, exosomes preconditioned with inflammatory stimuli exhibited reduced levels of NF-κB, IL-1β, and IFN-γ, demonstrating the ability of EVs to suppress key mediators of the acute inflammatory cascade (Shen et al., 2020) [40]. Finally, Yao et al. and Li et al. reported the downregulation of TGF-β in EV-treated groups, correlating with a decreased expression of collagen type III and α-SMA, suggesting that EV augmentation can diminish myofibroblast activity and matrix degradation during AT healing [31,37,49]. These results underscore EVs’ potential in modulating the inflammatory milieu to promote a conducive environment for AT repair. This aligns with emerging evidence endorsing stem cell-derived EVs for treating inflammatory conditions across diverse organ systems, including hepatic, pulmonary, and neural tissues [50].

### 4.2. Macrophage Polarization

An important process that signals the transition from the inflammatory phase to the proliferative phase of wound healing involves the phenotypic differentiation of M1 macrophages to M2 macrophages—a process known as macrophage polarization [51]. M1 macrophages, known for their role in early inflammatory response and debridement, can perpetuate a fibrotic response if left unchecked [52]. In fact, Zhang et al. found that M1 macrophages enhanced the expression of caspase-3 during tendon repair, thereby facilitating tenocyte apoptosis [38].

As the initial inflammation subsides, the macrophages transform into the anti-inflammatory M2 form via epigenetic modifications [53]. Anti-inflammatory cytokines, such as IL-4, augment this polarization process [51]. The resultant increase in M2 macrophages further stimulates IL-10, TGF-β, and IL-12, creating a positive feedback loop of anti-inflammatory changes [38,51]. Eight of the included studies found enhanced macrophage polarization to the M2 form within the EV treatment groups, indicating that it is likely a key molecular alteration by which EVs can improve AT healing. Moreover, Rong et al. found that treatment with MSC-derived exosomes decreased the M1/M2 macrophage ratio, resulting in endothelial cell proliferation [43]. This suggests that angiogenesis is one possible mechanism by which macrophage polarization can enhance wound healing. Other proposed mechanisms for how M2 macrophages can improve tissue repair include increased levels of vascular-specific growth factors, enhanced platelet recruitment to the injury site, and the induction of collagen fibril assembly [54,55,56].

### 4.3. Tenocyte Proliferation and Collagen Deposition

The proliferative phase of wound healing in tendon injury largely involves profound tenocyte proliferation and the resultant synthesis and deposition of collagen. Tenocytes are cells that primarily secrete collagen and maintain ECM homeostasis, and they account for roughly 18% of the volume in a tendon [57,58]. The ECM makes up the majority of the tendon and is mostly composed of water, type 1 collagen, and type 3 collagen [58,59]. It is also rich in proteoglycans, glycosaminoglycans, elastin, and other inorganic compounds.

Tendon injuries treated with EVs have been shown to increase tenocyte proliferation through effects on multiple different genes. Wang et al., Yao et al., and Chen et al. observed the increased expression of Tenomodulin (TNMD), a biomarker of matured tendinous tissue, with EV treatment [9,24,32]. The loss of TNMD has been associated with accelerated age-related tendon degeneration [60,61]. Yao et al. showed an almost two-fold increase in the expression of Scleraxis Homolog A (SCXA), a transcription factor that encourages tenocyte differentiation via increased TNMD activation [32,62,63]. In fact, SCXA-knockout mice have demonstrated an inability to transmit force through the affected tendons [64]. Furthermore, Liu et al. concluded that exosome treatment increased tenocyte proliferation through the upregulation of Mkx—another regulator of tenogenic differentiation [41]. The significance of the *Mkx* gene in AT wound healing is underscored by one study in which *Mkx*-knockout mice experienced ectopic ossification of their Achilles tendons within 1 month postnatally [65]. Interestingly, reductions in the heterotopic ossification of the injured tendons post-EV treatment were reported by Xu et al., but *Mkx* gene expression was not measured [34].

Increased tenocyte proliferation leads to greater collagen production and more type 1 collagen deposition [66]. In healthy tendinous tissue, the predominant form of collagen is type I collagen, which has thick collagen fibrils and is responsible for tensile strength and structural integrity [67]. Meanwhile, the amount of type III collagen, which has thinner collagen fibrils, in a healthy tendon is low [67,68]. Tendon injury often leads to disproportionate ratios of type 3 to type 1 collagen deposition, which has been associated with increased fibrosis, accelerated tendon aging, and disorganized collagen fiber orientation [30,67]. Ten of the studies in this review found increased ratios of type 1 to type 3 collagen deposition post-EV treatment. The proposed mechanisms for these findings include increased tenocyte proliferation (Rong et al. and Yao et al.) as well as the upregulation and downregulation of the *Col1a1* and *Col3a1* genes, respectively (Zhang et al. and Liu et al.) [31,38,41,43].

Additionally, the physical structure of the collagen was found to be improved in 14 of the 17 included studies that histologically analyzed the Achilles tendons (Supplementary Figure S2). Liu et al. and Chen et al. concluded that reductions in infiltrating inflammatory cells and edematous collagen cells led to optimized collagen organization [9,41]. The analysis conducted by Wellings et al. and Rong et al. revealed a denser and more uniform alignment of collagen fibers [30,43]. Similarly, Yao et al. and Xu et al. both described a more “spindle-like” arrangement of collagen fibrils, suggesting an increased tensile strength [24,39,69]. Meanwhile, Xu et al. and Hayashi et al. showed that EV treatment increased the collagen fibril diameter, demonstrating their potential in increasing tendon elasticity [34,39,70].

Superior collagen structure in tendons has also been associated with greater biomechanical properties [71]. Of the 11 studies that measured biomechanical characteristics, 10 found improved functionality with EV treatment (Supplementary Figure S2). Li et al. was the only study that did not find a difference in the biomechanical properties [37]. At least five of the studies [30,31,32,34,39] found an improved tensile strength, and four of the studies [22,30,36,39] reported an increased elastic modulus. These results indicate that EV treatments can help optimize the restoration of functional properties such as withstanding mechanical force, the transmission of contraction force from muscle to bone, and articular stability [69,72].

### 4.4. Matrix Metalloproteinases

Matrix metalloproteinases (MMPs) are a group of enzymes that regulate the ECM by degrading its components and play a pronounced role in the remodeling phase of wound healing. A critical aspect of tendon healing is the dynamic interplay between MMPs and tissue inhibitors of metalloproteases (TIMPs) [73]. EVs have been shown to optimize healing by modulating this balance. Some of the prominent MMPs involved in musculoskeletal disease are MMP-1, MMP-3, MMP-9, MMP-13, and MMP-14 [73]. MMP overactivity is often precipitated by inflammation, elevated thermal exposure, and mechanical stressors, which are factors that render tendons particularly vulnerable to injury [74,75,76,77]. In fact, MMP inhibitors have shown success in treating patients with Achilles tendinopathy [78,79]. Our observations suggest that EVs can modulate MMP activity post-AT injury and attenuate excess peritendinous fibrosis as a result.

Shen et al. demonstrated a downregulation of MMP-1, a type of collagenase that is induced by inflammation, after administering EVs to the injury site [40]. Given the ability of MMP-1 to degrade multiple different types of proteoglycans, including those that are integral to tendon mechanics such as aggrecan, the suppression of its activity can preserve normal ECM structure [80,81]. In fact, Shi et al. found a direct link between EV treatment and upregulated aggrecan expression, which also correlated with enhanced biomechanical properties such as force resistance, tensile strength, and elasticity [36].

Moreover, the studies conducted by Wang et al. and Liu et al. indicate that MMP-3, which has a broad substrate specificity and can degrade a variety of ECM components, is another pro-fibrotic enzyme that EVs can downregulate in the context of AT healing [32,41,82]. These findings align with other studies that have proposed that stem cell-derived EVs, via the downregulation of MMP-1 and MMP-3, can mitigate UVB-induced skin aging and corneal injury-induced visual impairments [83,84]. The expression of MMP-13—another collagenase that is often implicated in bone remodeling—was found to be decreased by Liu et al. after the administration of EVs post-tendon injury [41,80]. Similarly, Shen et al. proposed that EVs counteract MMP-13 and MMP-3 activity via the upregulation of reparative genes such as *Sox9* and *Col2a1* [40]. Interestingly, Chen et al. observed that rats in the EV treatment groups displayed elevated levels of two different types of proteoglycans, decorin and biglycan, which are often degraded by MMP-3 and MMP-13 in the EV treatment group [9,85,86]. In fact, the ability of EVs to protect against ECM hydrolysis by inhibiting MMP-13 expression has also sparked interest in their potential use in osteoarthritis therapies [87].

Additionally, MMP-9 and MMP-14 have emerged as potential targets in EV-mediated tendon healing [88]. MMP-9, known for its crucial role in angiogenesis, is in constant balance with TIMP-1. While MMP-9 actively degrades extracellular matrix components during inflammatory responses, TIMP-1 serves to inhibit this activity [73]. Following EV treatment, Zhang et al. observed a downregulation in MMP-9 coupled with an increase in TIMP-1 [38]. This suggests a more balanced ECM remodeling, tilting towards regeneration rather than degradation. Conversely, MMP-14, another collagenase that is integral to angiogenesis, exhibited increased expression post-EV application in the study conducted by Gissi et al. [42]. This elevation in MMP-14 levels implies a potential enhancement in the reparative processes, a unique aspect of EV therapy in contrast to the suppression of other MMPs. Notably, MMP-14 is also known to degrade fibronectin, which is a pro-fibrotic ECM glycoprotein [73]. Zhang et al. also found that EV treatments decreased fibronectin expression [38]. Collectively, these findings illuminate the nuanced and multifaceted role of EVs in modulating various MMPs, aligning ECM degradation and synthesis towards efficient tendon healing.

### 4.5. MicroRNAs

MicroRNAs (miRNAs) are small, non-coding RNA molecules that play a key role in the post-transcriptional regulation of gene expression [89]. miRNAs are typically 19–25 nucleotides in length and act primarily through base-pairing interactions with the 3′ untranslated region (3′ UTR) of target mRNAs in the cell cytoplasm, leading to translational repression, mRNA degradation, or chromatin remodeling [90,91,92]. Their abundant presence within EVs has made them a topic of great interest in the study of EVs in the context of accelerated wound healing and the identification of diagnostic biomarkers for numerous diseases. Our review has identified five major families of miRNAs that were found to have an effect on AT healing: miR-21, miR-27b, miR-29a, miR-146a, and miR-148a.

The miR-21 family is one of the most studied miRNAs and has been associated with diseases such as metastatic tumors of the breast, lung, prostate, and liver [93]. It has also been shown to have pro-fibrotic effects on multiple organ systems, including cardiac, pulmonary, and renal tissues [94,95,96]. However, the role of miR-21 is less clear in the context of tendon healing. Yao et al. found that the subcutaneous injection of HUMSC-Exos into rat ATs decreased the expression of miR-21a-3p, inducing the inhibition of the p65 and COX2 genes, both of which have been associated with increased fibrosis in other organs such as the lung and liver [31,97,98]. This inhibition of p65 and COX-2 led to the downstream inhibition of COL III and α-SMA in rat tendons, suggesting a mechanistic pathway by which EVs can reduce fibrosis via miR21 modulation in the context of tendon healing [31]. Conversely, Xu et al. found that miR-21-5p was upregulated in ASC-Exos, and thus associated with greater tendon healing when compared to the ASC-derived microvesicle group [39]. This finding suggests that the upregulation, rather than downregulation, of the miR-21 family could stimulate AT healing. Conflicting results regarding the effects of miR-21 on tendon healing are present in the existing literature as well: Thankam et al. found that the downregulation of miR-21-5p was associated with disorganized collagen deposition in human biceps tendons, while Cui et al. demonstrated that the upregulation of miR-21-5p through BMSC-derived exosomes led to increased fibrosis and tendon adhesions [99,100]. Although it seems to be widely agreed upon that the miR-21 family of miRNAs has negative effects on many other organ systems, further research needs to be conducted to elucidate its role in tendon healing specifically.

The miR-29 family, on the other hand, has been widely accepted as a pro-healing agent, not only in tendons but in other cell types as well. In tendons, the downregulation of miR-29a has been shown to increase Collagen III synthesis and decrease Collagen I synthesis—a process that seems to be mediated by IL-33 [101,102]. Another study proposed that miR-29 can also contribute to the maintenance of tendon health via the regulation of bone morphogenetic protein (BMP)-2, BMP-7, and IL-6 [103]. Apart from tendons, the upregulation of miR-29 has been shown to reduce muscular atrophy and renal fibrosis via the downregulation of α-SMA, fibronectin, and COL I. Moreover, deficiencies in certain variants of miR-29 have been linked to immunological conditions such as multiple sclerosis and various types of lymphomas [104,105]. Our observations suggest that miR-29 has a similarly positive impact on AT healing. Yao et al. demonstrated that HUMSC-Exos that were engineered to overexpress miR-29a-3p via specific miRNA agonists improved the organization of collagen fibers in rat ATs [24]. Their findings also highlighted an increased level of phosphorylated mTOR, hinting that the slowing of age-related changes in injured ATs is a possible mechanism through which EVs and miRNAs can promote healing. Xu et al. also found that exosomes with an elevated expression of miR-29a were more likely to have reduced Collagen III expression [39]. The collective findings from these studies position miR-29a as a pivotal miRNA in the context of AT healing and potentially offer a promising avenue for future therapeutic interventions.

Other miRNAs were also found to be effective. Han et al. found that a single subcutaneous injection of HUMSC-Exos upregulated the expression of miR-27b-3p, resulting in increased tenocyte proliferation and the invasion of injured tenocytes [36]. The proposed mechanism by which this occurs is due to the ability of miR-27b-3p to suppress the *Arhgap5* gene, which is a well-known inhibitor of RhoA activity. Thus, by increasing RhoA activity, tendon healing was enhanced as well, a finding that was consistent with other wound healing studies involving the skin, colon, and airway tracts [106,107,108]. Although increased cell proliferation can be a positive result in the context of wound healing, other studies have suggested that the excessive upregulation of RhoA activity can also have tumorigenic or pro-senescent effects, thus indicating the need for further research to clarify its role in tendinous healing [109,110]. Furthermore, Xu et al. also found that ASC-Exos treatment led to theupregulation of miR-148a, which has been associated with improved angiogenesis via the upregulation of the thrombospondin-4 gene and the downregulation of Kruppel-like factor 6 (KLF6) [39,111,112]. However, it is unclear whether, instead of inducing tendinous healing, miR-148a can propagate excess angiogenesis and thus induce a pathological state of tendinopathy [111,112]. Lastly, Shen et al. concluded that the conversion of M1 to M2 macrophages that was observed after priming ASC-Exos with IFN-γ could be attributed to the upregulation of miR-146a—a response that has been observed by previous studies as well [40,113,114]. Additionally, it has been suggested that, like miR-148a, miR-146a can also enhance angiogenesis by targeting and inhibiting the anti-angiogenic vasohibin-1 (*Vash1*) gene; however, whether this can facilitate tendon healing is unclear and requires further research [112,115].

### 4.6. Mechanism of Delivery

The most common methods of delivery were the injection of stem cell or platelet-derived EVs or the implantation of EV-laden scaffolds at the site of injury. Although these methods are widely used and largely successful, they have been associated with poor cell survival and excessive inflammatory responses at times [114]. To combat these potential limitations, the use of biomaterials composed of various polymers or magnetic elements to mechanically stimulate cells have gained increasing popularity as solutions to further optimize tissue regeneration [116]. Three of the included studies have utilized unique biomaterials to deliver EVs to the injured ATs.

First, Xu et al. prepared a bioactive glass (BG) made up of 45% SiO_2_, 24.5% Na_2_O, 24.5% CaO, 6% P_2_O_5_, and GelMA composite as a carrier for MSC-derived EVs to facilitate tendon repair [34]. The GelMA was fully dissolved in PBS and blended with a photo-initiator, creating a pre-polymer solution that was subsequently loaded with the EVs. Pretreatment with EV-laden GelMA scaffolds like this one have been shown to influence intercellular interaction via augmented paracrine signaling activities [117,118,119]. Meanwhile, Rong et al. created an organic, uniform Zinc-based nanocube, known as an enzymatic nanocube (EN), through the coordination of Zn^2+^ ions, dimethylimidazole ligands, RuCl_3_ solution, and NaBH [43,120]. The isolated exosomes were cloaked by the ENs via co-incubation with shaking, forming ENEVs. Following the injection of ENEVs, low-frequency acoustic waves were generated at the injury site via ultrasound, exerting a sonoporation effect on the damaged cells, thus enhancing the endocytic uptake of exosomes. Lastly, Liu et al. enriched isolated exosomes with a collection of 2-methacryloyloxyethyl phosphorylcholine (MPC) monomers that coated the exosomes after polymerizing around them [41]. L-arginine was subsequently added onto this polymer coat, resulting in the formation of what was termed a nanomotor. These nanomotors were loaded into a microneedle array, which penetrated the skin into the superficial dermis. The L-arginine uses the inflammation-induced ROS and nitric oxide synthase to drive the exosomes deeper into the injured tissue where the polymer coat degrades and exosomes are released [41].

The results of the included studies suggest improved wound healing in a variety of ways (Table 3), with a commonality being the enhanced modulation of collagen secretion and more organized collagen fiber alignment. These results suggest that when delivering EVs, achieving deeper tissue penetration and/or less obstructed access to the injury site can significantly augment tenocyte proliferation. The mechanism of delivery is likely an important determinant of the therapeutic potential of EV treatments, though more research is required to determine optimal delivery methods.

### 4.7. Clinical Relevance of EVs

The presence of EV-based therapies is rapidly increasing on the clinical scene. Although there are no FDA-approved EV treatments for clinical use at this time, dozens of EV-based clinical trials are currently being conducted. EVs are being investigated for two primary clinical uses. The majority of such clinical trials underway are assessing the microRNA profiles of EVs in various organs to improve diagnostic ability for many types of cancers, including but not limited to melanoma, prostate cancer, and other cancers and metastatic lesions within the lungs [121,122,123,124]. Beyond cancer, some clinical trials are even assessing if various exosome-based miRNAs, such as miR-136, can be used as biomarkers to aid in the diagnosis of conditions such as preeclampsia and type 2 diabetic nephropathy [125,126].

However, a handful of clinical trials underway are also investigating the potential of EVs as a treatment modality for both wound healing and disease resolution. A clinical trial being conducted by Shanghai Jiao Tong University is testing the ability of a topical exosome-hydrogel scaffold on wound healing. Furthermore, a clinical trial being conducted by the Himanshu Bansal Foundation (NCT04849429) is evaluating exosome-enriched platelet-rich plasma injections for their use in chronic back pain associated with intervertebral disc pathology [127,128]. It is important that EVs can be derived from the cells of any eukaryotic organism. In fact, there are multiple ongoing clinical trials that are assessing the ability of plant-based exosomes to attenuate inflammation in conditions such as inflammatory bowel disease and chemoradiation-associated mucositis [129,130]. Another interesting research focus regarding the clinical application of EVs that has yielded positive outcomes thus far is for patients with coronavirus-associated acute respiratory distress syndrome and pneumonia [131,132,133].

The burgeoning field of EV-based therapies holds promise across a spectrum of diseases, from diagnostic advancements to innovative treatments. As clinical trials progress and more data emerges, the scope for EVs in medical applications may expand, potentially offering novel therapeutic avenues where traditional treatments fall short.

However, several significant limitations of EV technology warrant careful consideration. One major challenge is the reproducibility of results across different studies and clinical trials. Variations in EV isolation methods, donor cell conditions, and characterization techniques can lead to inconsistencies in EV quality and function, and thus affect outcome reproducibility. Another concern involves regulatory approval, as the therapeutic use of EVs falls into a complex regulatory framework that is still evolving. Stringent standards for safety, efficacy, and quality control must be met to gain regulatory approval. Additionally, even upon regulatory approval, the potential for scale-up remains a concern. Transitioning from laboratory-scale production to the large-scale manufacturing of EVs presents logistical and technical challenges that could impede their clinical application. Collectively, these factors highlight the need for standardized methodologies and more robust regulatory guidelines to fully realize the clinical benefits of EV technologies.

### 4.8. Limitations

In this systematic review, several limitations warrant consideration. First, we are unable to draw definitive conclusions from the observations made in this review due to the heterogeneity in experimental design, outcomes, species, and measurement of results. There also exists the potential for publication bias, wherein studies with positive findings are more likely to be published than those with negative or inconclusive results, potentially skewing the therapeutic efficacy of EVs. Additionally, the methodological quality across the included studies varied, introducing the possibility of bias in the reported outcomes, and thus affecting the overall conclusions drawn from this review. Moreover, the small sample size in many of the studies may limit the generalizability and robustness of the findings. Lastly, the possibility of evidence selection bias should be considered, as studies not indexed in the PubMed, Scopus, and Embase databases may have been inadvertently excluded.

It is also essential to address the inherent anatomical and physiological differences between human and rodent/rabbit ATs. Rodent models offer valuable insights due to some shared characteristics with humans, as ATs from rodents originate from the same three calf muscles as those from humans and have comparable relative sizes [134]. Also, concerning the profile and activity of inflammatory signaling molecules in tendinous wound healing, rat tenocytes closely mirror human tenocytes [135]. However, their ATs also differ from those of humans in terms of certain anatomical properties, such as lacking fascicles and interfascicular matrix, which can lead to marked differences in the healing process relative to humans [135]. These discrepancies must be considered when extrapolating these findings to human AT healing.

## 5. Conclusions

The observations of our systematic review provide strong preliminary evidence within the literature, which support the notion that stem cell-derived EVs can accelerate tendon healing without posing any serious risk of adverse effects. However, only in vivo animal studies have been conducted to this point. More research, including clinical trials on human subjects, is needed to elucidate the various mechanisms by which EVs promote healing as well as to better calculate their therapeutic potential. Ultimately, AT pathologies can prove difficult to treat, and often include long, difficult recoveries. Novel treatment strategies are required in the field of tendon healing. EVs are emerging as a promising therapeutic option that can not only help usher in a new era of tendon and connective tissue treatments and improve clinical outcomes, but also deepen our fundamental understanding of drug delivery, diagnostic techniques, and wound healing.

## Figures and Tables

**Table 1:** Characteristics of included studies, type of model used, and SYRCLE risk of bias outcome.

Study (Year)	Journal	Country	Risk Assessment	In Vivo Model
Yao et al. (2020) [31]	*Journal of Intrinsic Inflammation*	China	Low	Rat
Wellings et al. (2021) [30]	*Orthopedic Journal of Sports Medicine*	USA	Low	Rabbit
Wang et al. (2019) [32]	*Journal of Cellular and Molecular Medicine*	China	Medium	Rat
Hayashi et al. (2022) [33]	*FEBS Journal*	Japan	Low	Mouse
Yao et al. (2021) [24]	*Journal of Nanotechnoloft*	China	Low	Rat
Xu et al. (2023) [34]	*Biomaterials*	China	Low	Rat
Chamberlain et al. (2019) [22]	*Stem Cells*	USA	Medium	Mouse
Han et al. (2022) [35]	*Acta Biochimica et Biophysica Sinica (ABBS)*	China	Low	Rat
Shen et al. (2023) [23]	*Stem Cells*	USA	Low	Mouse
Shi et al. (2020) [36]	*Medical Science Monitor*	China	Medium	Mouse
Li et al. (2020) [37]	*Stem Cell Research & Therapy*	China	Medium	Rat
Zhang et al. (2020) [38]	*Stem Cell Research & Therapy*	China	Low	Rat
Xu et al. (2022) [39]	*The American Journal of Sports Medicine*	China	Low	Rat
Shen et al. (2020) [40]	*Journal of Orthopedic Research*	USA	Low	Mouse
Liu et al. (2021) [41]	*ACS Nanotechnology*	China	Medium	Rat
Gissi et al. (2020) [42]	*PLOS ONE*	Italy	Low	Rat
Rong et al. (2023) [43]	*ACS Nanotechnology*	China	Medium	Rat
Chen et al. (2021) [9]	*International Journal of Molecular Sciences*	Taiwan	Low	Rabbit

**Table 2:** Extracellular vesicle type, cell of origin, mechanism for Achilles tendon injury, and method for delivery of extracellular vesicles.

Study Type	EV Type	Cell Origin	Tendon Injury Mechanism	Method of Delivery	Experimental Group	Positive Control	Negative Control
Yao et al. (2020) [31]	Exosomes	Human UCSCs	Incision made in middle and deep Achilles tendon	EVs were injected subcutaneously around injury site.	hUMSC-Exos group (*n* = 20)	-	1. PBS-only group (*n* = 20); 2. surgical repair-only group (*n* = 20)
Wellings et al. (2021) [30]	Exosomes	Activated platelets	Achilles tendon tenotomy proximal to calcaneal tubercle	Total of 0.2 mL of scaffold was placed topically at tenotomy site followed by tightening of suture.	Type 1 collagen scaffold loaded with 20% PEP group (*n* = 15)	Type 1 collagen scaffold-only group (*n* = 15)	Surgical repair-only group (*n* = 15)
Wang et al. (2019) [32]	Exosomes	Rat TSCs	Collagenase injections of Achilles tendons (micro-damaged model)	Both injury groups were injected with exosomes or TSCs into L tendon while PBS was injected into R tendon.	Injury group with exosomes treatment (*n* = 6)	Injury group with TSCs treatment (*n* = 6)	PBS-only group *(n* = 6)
Hayashi et al. (2022) [33]	Exosomes	Human BMSCs	Complete transverse incision was made at midpoint of Achilles tendon	Amount of 20 µL of P5 and P12 MSC-EV suspension with PBS was injected into gap between transected tendons at 1 and 7 days post-transection.	1. Early passaged (P5) BMSC-EVs (*n* = 5); 2. senescent (P12) BMSC-EVs (*n* = 5)	-	PBS-only group (*n* = 5)
Yao et al. (2021) [24]	Exosomes	Human UCSCs	Full-thickness defect of Achilles tendon	Implantation with fibrin glue at tendon injury site.	50 μL fibrin glue + 100 μg of Human UMSCs with Exosomes (HUMSC-Exos) (*n* = 30)	50 μL fibrin-only group (*n* = 30)	Surgical repair-only group (*n* = 30)
Xu et al. (2023) [34]	Exosomes	Human ATSCs	Full-thickness transection near proximal end of calcaneal insertion of Achilles tendon	Prior to completing surgical repair, 20 μL of GelMA hydrogel or EV-laden GelMA hydrogel was implanted between transected tendon margins.	1. BioGlass-elicited ATSC-EV-laden GelMA hydrogel group (*n* = 20); 2. naïve ATSC-EV-laden GelMA hydrogel group (*n* = 20)	GelMA hydrogel-only implantation group (*n* = 20)	Tendon intact group (*n* = 20); surgical repair-only group (*n* = 20)
Chamberlain et al. (2019) [22]	Exosomes	Human BMSCs	Full-thickness transection was made at midpoint of Achilles tendon	Tendon margins were sutured together, and using muscular layer, surgical pouch was created over injury site. Treatments with 20 μL were administered to injury site using this surgical pouch.	EV/exosome-educated macrophage group (*n* = 27 among all groups)	2. MSC-only group	1. Control macrophage group; 2. daline-only group (*n* = 27 among all groups)
Han et al. (2022) [35]	Exosomes	Human UCSCs	Superficial Achilles tendon was removed, and transverse midline cut was made in middle of deep Achilles tendon	Single subcutaneous injection of HUMSC-Exos (100 μg) was administered at injury site.	hUMSC-Exos group (*n* = 6)	-	Surgical repair-only group (*n* = 6); model group without injury (*n* = 6)
Shen et al. (2023) [23]	Exosomes	Mouse ATSCs	Midline transection of Achilles tendon between calcaneal insertion and myotendinous junction	Collagen sheet was pre-loaded on one side with iEVs, which was applied around repair site with iEV-side facing repaired tendon.	1. Type 1 collagen sheet loaded with 5 × 10^9^ inflammation-primed ATSC extracellular vesicles (iEVs) (*n* = 11); 2. type 1 collagen sheet loaded with 1 × 10^9^ iEVs (*n* = 11)	Type 1 collagen sheet-only group (*n* = 10)	-
Shi et al. (2020) [36]	Exosomes	Mouse BMSCs	Cut was induced in Achilles tendon at bone–tendon junction with calcaneus	Hydrogel and hydrogel-BMSC-Exos were implanted at bone–tendon junction injury site.	Hydrogel with exosome group (*n* = 30)	Hydrogel-only group (*n* = 30)	Surgical repair-only group (*n* = 30)
Li et al. (2020) [37]	Exosomes	Human UCSCs	Thin strand of Achilles tendon was resected via “S incision” and midline transection was inflicted on deep Achilles tendon	Subcutaneous injection with 50 μL of HCPT-EVs or unprimed EVs at injury site after wound closure and suture repair.	Hydroxycamptothecin-primed human UCSCs in EV group (HCPT-EVs) (*n* = 11)	Unprimed EVs group (*n* = 11)	PBS-only group (*n* = 11)
Zhang et al. (2020) [38]	Exosomes	Rat TSCs	One-third of central part of Achilles tendon was removed	TSC-Exos and GelMA-only groups were placed in Achilles tendon defect and irradiated into gel state via 10–20 s of exposure to blue light source (405 nm) at 3 cm away.	TSC-Exos group with GelMA (*n* = 18)	TSC-Exos group (*n* = 18)	GelMA-only group (*n* = 18)
Xu et al. (2022) [39]	Exosomes and Microvesicles	Rat ATSCs	Vertical incision was made bilaterally in Achilles tendon	Vertical incision was made in Achilles tendon. One week later, in 24 rats, 10^10^ exosomes were suspended in 25 µL of saline in L Achilles tendon and 10^10^ ectosomes were suspended in 25 µL of saline in R Achilles tendon.	1. ATSC-Exos group (*n* = 24); 2. ATSC-Ectos group (*n* = 24)	-	Saline-only group (*n* = 12)
Shen et al. (2020) [40]	Exosomes	Mouse ATSCs	Two-thirds transection was inflicted at midpoint level of Achilles tendon	EV-laden collagen sheet was cut into strips and applied around repair site.	Collagen sheet loaded with EVs from IFNγ-primed ASCs (+iEVs) group (*n* = 10)	Collagen sheet loaded with EVs from naïve ASCs (+EVs) group (*n* = 11)	Collagen sheet-only group (*n* = 11)
Liu et al. (2021) [41]	Exosomes	Rat TSCs	Collagenase I injections performed to establish state of Achilles tendinopathy	Rats in Exos/MBA and Exos groups were given equal injections of Exos/MBA-loaded microneedle arrays and Exos only at site of injury, respectively.	Exosome delivered with nitric oxide nanometer group (*n* = 5)	Exosome-only (EXO) group (*n* = 5)	1. Achilles tendinopathy only group (*n* = 5); 2. injury-only group (*n* = 5)
Gissi et al. (2020) [42]	Extracellular Vesicles (not specified)	Rat BMSCs	Incision in Achilles tendons	Not stated.	1. High concentration of rat bone marrow MSC group (*n* = 4); 2. low concentration of rBMSC-EV group (*n* = 4)	rBMSC-only group (*n* = 4)	PBS-only group (*n* = 4)
Rong et al. (2023) [43]	Exosomes	Rat BMSCs	Midline transection of Achilles tendon	EN, EV, and ENEV groups involved injections at injury site. For ENEV-US group, ENEV was injected at injury site and was immediately followed by ultrasound irradiation of injury site to augment cellular uptake.	EV-cloaked enzymatic nanohybrid (ENEV) group with ultrasound irradiation (ENEV-US) group (*n* = 10)	1. EN-only group (*n* = 10); 2. EV-only group (*n* = 10); 3. ENEV group (*n* = 10)	PBS-only group (*n* = 10)
Chen et al. (2021) [9]	Extracellular Vesicles (not specified)	Rabbit ATSCs	Achilles tendon transection via longitudinal superficial incision	EV solution was injected into tendon at injury site.	ATSC-EV group (*n* = 18)	-	PBS-only group (*n* = 18)

Abbreviations: UCSCs = umbilical cord stem cells; TSCs = tendon stem cells; BMSCs = bone marrow stem cells; ATSCs = adipose tissue stem cells; PBS = phosphate-buffered saline; PEP = purified exosome product; GelMA = gelatin methacrylate; Exos = exosomes; MBA = nitric oxide nanometer; EV = extracellular vesicle; EN = enzymatic nanohybrid.

**Table 3:** Suggested mechanisms by which extracellular vesicles exert therapeutic influence for all included studies.

Study Type	Regulation of Inflammation and Immune Response	MicroRNA Regulation	Macrophage Polarization	Gene Regulation of ECM Components	Cell Proliferation and Migration
Yao et al. (2020) [31]	↓ TGF-β, ↓ α-SMA, ↓ p-65, ↓ COX-2	↓ miR-21a-3p	-	↓ COL III	↓ fibroblast proliferation
Wellings et al. (2021) [30]	-	-	-	-	-
Wang et al. (2019) [32]	-	-	-	↑ *Col1a1*, ↑ TIMP-3, ↑ Tenomodulin ↓ MMP-3	-
Hayashi et al. (2022) [33]	-	-	-	-	-
Yao et al. (2021) [24]	-	↑ miR-29a-3p	-	↑ *Col1a1*, ↑ SCXA, ↑ Tenomodulin	↓ mTOR
Xu et al. (2023) [34]	-	↑ miR-199b-3p, ↑ miR-125a-5p	↑ M2	-	-
Chamberlain et al. (2019) [22]	-	-	↓ M1/M2 ratio	-	↑ angiogenesis
Han et al. (2022) [35]	-	↑ miR-27b-3p	-	-	↑ RhoA, ↓ ARHGAP5
Shen et al. (2023) [23]	↑ *Arg1*, ↑ IL-13 ↓ IL-1β, ↓ TLR4/NF-kB	↑ miR-147-3p	↓ M1/M2 ratio	-	-
Shi et al. (2020) [36]	↑ IL-10, ↑ TGF-β1, ↓ IL-1β, ↓ IL-6	-	↑ M2, ↓ M1	↑ COL II, ↑ Aggrecan	↑ TGF-β3, ↑ IGF-1, ↑ IGF-2, ↑ CD146
Li et al. (2020) [37]	-	-	-	↓ COL III, ↓ α-SMA	↑ Bax, ↑ fibroblast proliferation, ↑ myofibroblast differentiation, ↓ Bcl-2
Zhang et al. (2020) [38]	↑ IL-10, ↓ IL-6, ↓ COX-2	-	↑ M2, ↓ M1	↑ TIMP-1, ↑ *Col1a1*/*Col3a1* ratio, ↓ α-SMA, ↓ fibronectin, ↓ MMP-9	↑ AKT, ↑ ERK1/2
Xu et al. (2022) [39]	-	↑ miR-29a, ↑ miR-21-5p, ↑ miR-148a-3p	-	↑ COL I, ↓ COL III	↑ tenocyte proliferation and migration, ↑ angiogenesis
Shen et al. (2020) [40]	↓ NF-kB, ↓ IL-1β, ↓ IFN-y	↑ miR-let-7b, ↑ miR-146a	↓ M1/M2 ratio	↑ *Col2a1*, ↑ *Sox9*, ↓ *Mmp1*, ↑ COL I/COL III ratio	-
Liu et al. (2021) [41]	↓ IL-1β, ↓ IL-6, ↓ IL-8, ↓ IL-18, ↓ iNOS, ↓ CXCL	-	-	↑ Col1a, ↓ Col3, ↓ MMP-3, ↓ MMP-13	↑ *Mkx*, ↑ EdU, ↑ PCNA
Gissi et al. (2020) [42]	-	-	-	↑ MMP-14, ↑ pro-collagen1A2	surface proteins ↑, tenocyte proliferation and migration
Rong et al. (2023) [43]	↓ IL-1β, ↓ IL-6, ↓ TNF-α	-	↑ M2	↑ COL I	↑ tenocyte proliferation
Chen et al. (2021) [9]	-	-	-	↑ COL I, ↑ tenomodulin, ↑ biglycan, ↑ decorin	↑ tenomodulin

Abbreviations: TGF-β = transforming growth factor-β; α-SMA = α-smooth muscle actin; miR = microRNA; MMP-x = matrix metalloproteinase-x; TIMP-y = tissue inhibitor of metalloproteinase-y; IL-z = interleukin-z; M1 = M1 macrophage; M2 = M2 macrophage; TNF-α = tumor necrosis factor-α; COL x = type x collagen. Symbols: up arrow denotes increased expression of gene or production of protein; down arrow denotes decreased expression of gene or production of protein.

## Supplementary Materials

The following supporting information can be downloaded at: https://www.mdpi.com/article/10.3390/biomedicines12050942/s1, Figure S1: Heatmap representation of specific biomechanical and histological results for included studies. Table S1: PRISMA Checklist for Systematic Reviews. Table S2: Proposed overall results, clinical applications, and future studies by the included studies.
