# Supplementary material for: The Use of Extracellular Vesicles in Achilles Tendon Repair: A Systematic Review
Source: Biomedicines. 2024 Apr 23;12(5):942. doi: 10.3390/biomedicines12050942 (PMC11117955; doi:10.3390/biomedicines12050942)
Supplement: Supplementary file 1 [file biomedicines-12-00942-s001.zip › biomedicines-2949362-supplementary.pdf]

Supplementary Materials

2. Materials and Methods

2.1. Search Criteria and Screening Strategy

A database search was conducted in October 2023 in PubMed, Scopus, and Embase using various combinations of the terms including: "Achilles tendon, Achilles tendon repair, Achilles tendon surgery, extracellular vesicle, extracellular vesicle, exosome, exosomes, microvesicle, microvesicles, ectosome, ectosomes, apoptotic body, apoptotic bodies." Search results were imported into Rayyan, a free online software used for systematic reviews, where they were deduplicated [1]. Using Rayyan, articles were screened by two independent and blinded reviewers (VK and VP) and conflicts were resolved by a third independent reviewer (NG). Articles were included based on if they met the following inclusion criteria: 1) Must be a full text paper that was published in a peer-reviewed journal; 2) Studies involving human or animal models; 3) Studies that investigate the use of extracellular vesicles or exosomes in vivo as therapeutic intervention for Achilles tendon injuries; 4) Studies reporting relevant outcomes related to tendon healing, regeneration, or mechanistic insights.

The search strategy was conducted in accordance with PRISMA guidelines, including a flow chart (Figure 1) and checklist (Table S1) [2].

2.2. Data Extraction and Variables of Interest

Included articles were subjected to a manual data extraction process using Microsoft Excel (Version 16.84, Redmont, WA, USA). Variables regarding study characteristics such as country, journal, year, and animal model were collected. Further, information for experimental and control group parameters, mechanism of injury and treatment were also recorded. Finally, information was collected regarding treatment outcome, histological analysis, and proposed mechanism of action.

2.3. Quality Assessment

The Systematic Review Center for Laboratory Animal Experimentation (SYRCLE) tool was utilized to conduct a rigorous quality assessment of the included in vivo studies [3]. It consists of 10 different parameters and an overall risk of bias score (Figure 2). The quality assessment was independently completed by KB and VK, and all conflicts were resolved by a third independent reviewer NG.

The protocol of this review was recorded in the INPLASY register under the number INPLASY202430019.

Supplementary Table S1: PRISMA Checklist for Systematic Reviews

Section and Topic	Item #	Checklist item	Location where item is reported
TITLE			
Title	1	Identify the report as a systematic review.	Title
ABSTRACT			
Abstract	2	See the PRISMA 2020 for Abstracts checklist.	Done
INTRODUCTION			
Rationale	3	Describe the rationale for the review in the context of existing knowledge.	Done
Objectives	4	Provide an explicit statement of the objective(s) or question(s) the review addresses.	Done
METHODS			
Eligibility criteria	5	Specify the inclusion and exclusion criteria for the review and how studies were grouped for the syntheses.	Methods (section 2.1)
Information sources	6	Specify all databases, registers, websites, organisations, reference lists and other sources searched or consulted to identify studies. Specify the date when each source was last searched or consulted.	Methods (section 2.1)
Search strategy	7	Present the full search strategies for all databases, registers and websites, including any filters and limits used.	Methods (section 2.1)
Selection process	8	Specify the methods used to decide whether a study met the inclusion criteria of the review, including how many reviewers screened each record and each report retrieved, whether they worked independently, and if applicable, details of automation tools used in the process.	Methods (section 2.1)
Data collection process	9	Specify the methods used to collect data from reports, including how many reviewers collected data from each report, whether they worked independently, any processes for obtaining or confirming data from study investigators, and if applicable, details of automation tools used in the process.	Methods (section 2.1 & 2.2)
Data items	10a	List and define all outcomes for which data were sought. Specify whether all results that were compatible with each outcome domain in each study were sought (e.g. for all measures, time points, analyses), and if not, the methods used to decide which results to collect.	Methods & Results (section 2.2, tables 1-3)
	10b	List and define all other variables for which data were sought (e.g. participant and intervention characteristics, funding sources). Describe any assumptions made	Table 1

Section and Topic	Item #	Checklist item	Location where item is reported
		about any missing or unclear information.	
Study risk of bias assessment	11	Specify the methods used to assess risk of bias in the included studies, including details of the tool(s) used, how many reviewers assessed each study and whether they worked independently, and if applicable, details of automation tools used in the process.	Methods (section 2.3)
Effect measures	12	Specify for each outcome the effect measure(s) (e.g. risk ratio, mean difference) used in the synthesis or presentation of results.	N/A
Synthesis methods	13a	Describe the processes used to decide which studies were eligible for each synthesis (e.g. tabulating the study intervention characteristics and comparing against the planned groups for each synthesis (item #5)).	N/A
	13b	Describe any methods required to prepare the data for presentation or synthesis, such as handling of missing summary statistics, or data conversions.	Methods (section 2.3)
	13c	Describe any methods used to tabulate or visually display results of individual studies and syntheses.	N/A
	13d	Describe any methods used to synthesize results and provide a rationale for the choice(s). If meta-analysis was performed, describe the model(s), method(s) to identify the presence and extent of statistical heterogeneity, and software package(s) used.	Results (section 3.1)
	13e	Describe any methods used to explore possible causes of heterogeneity among study results (e.g. subgroup analysis, meta-regression).	Results (section 3.1)
	13f	Describe any sensitivity analyses conducted to assess robustness of the synthesized results.	Results (section 3.1)
Reporting bias assessment	14	Describe any methods used to assess risk of bias due to missing results in a synthesis (arising from reporting biases).	Methods (section 2.3)
Certainty assessment	15	Describe any methods used to assess certainty (or confidence) in the body of evidence for an outcome.	Methods (section 2.3)
RESULTS			
Study selection	16a	Describe the results of the search and selection process, from the number of records identified in the search to the number of studies included in the review, ideally using a flow diagram.	Results (section 3.1 & Figure 1)
	16b	Cite studies that might appear to meet the inclusion criteria, but which were excluded, and explain why they were excluded.	Figure 1

Section and Topic	Item #	Checklist item	Location where item is reported
Study characteristics	17	Cite each included study and present its characteristics.	Done
Risk of bias in studies	18	Present assessments of risk of bias for each included study.	Figure 2
Results of individual studies	19	For all outcomes, present, for each study: (a) summary statistics for each group (where appropriate) and (b) an effect estimate and its precision (e.g. confidence/credible interval), ideally using structured tables or plots.	N/A
Results of syntheses	20a	For each synthesis, briefly summarise the characteristics and risk of bias among contributing studies.	Figure 2
	20b	Present results of all statistical syntheses conducted. If meta-analysis was done, present for each the summary estimate and its precision (e.g. confidence/credible interval) and measures of statistical heterogeneity. If comparing groups, describe the direction of the effect.	N/A
	20c	Present results of all investigations of possible causes of heterogeneity among study results.	N/A
	20d	Present results of all sensitivity analyses conducted to assess the robustness of the synthesized results.	N/A
Reporting biases	21	Present assessments of risk of bias due to missing results (arising from reporting biases) for each synthesis assessed.	N/A
Certainty of evidence	22	Present assessments of certainty (or confidence) in the body of evidence for each outcome assessed.	N/A
DISCUSSION			
Discussion	23a	Provide a general interpretation of the results in the context of other evidence.	Discussion (section 4)
	23b	Discuss any limitations of the evidence included in the review.	Discussion (section 4.7)
	23c	Discuss any limitations of the review processes used.	Discussion (section 4.7)
	23d	Discuss implications of the results for practice, policy, and future research.	Conclusions (section 5)
OTHER INFORMATION			
Registration and protocol	24a	Provide registration information for the review, including register name and registration number, or state that the review was not registered.	Done
	24b	Indicate where the review protocol can be accessed, or state that a protocol was not prepared.	Done
	24c	Describe and explain any amendments to information	Done

Section and Topic	Item #	Checklist item	Location where item is reported
		provided at registration or in the protocol.	
Support	25	Describe sources of financial or non-financial support for the review, and the role of the funders or sponsors in the review.	Title page
Competing interests	26	Declare any competing interests of review authors.	Title page
Availability of data, code and other materials	27	Report which of the following are publicly available and where they can be found: template data collection forms; data extracted from included studies; data used for all analyses; analytic code; any other materials used in the review.	Done

Supplementary Table S2: Proposed overall results, clinical applications, and future studies by the included studies/

Study (Year)	Study Results	Proposed Implications/Applications for Clinical Use	Proposed Future Studies
Yao et al. (2020) [31]	Administration of human UMSC-Exos reduced fibroblast proliferation, alleviated tendon adhesion, as well as lowered expression of pro-fibrotic factors like collagen type 3 and a-smooth muscle actin.	-	Further characterization of the interaction between miR-21 and p-65 is needed.
Wellings et al. (2021) [30]	Administration of PEPs restored mechanical function in tendon injury despite smaller cross-sectional area than control in AT injury. PEPs also inhibited adhesion formation.	Application in chronic tendon disease due to promotion of intrinsic tendon healing.	Include stains of tenomodulin and decorin. Analyze effects on inflammatory cascade.
Wang et al. (2019) [32]	TSCs and exosomes derived from TSCs promoted tendon healing and tendon matrix maintenance. They decreased expression of MMP-3, and increased expression of TIMP-3 and Col-1a1. They both increased	This is a new pathway for tendon injury drugs to target as well as a new mechanism for delivering these drugs.	-

	biomechanical properties like maximum loading as well.		
Hayashi et al. (2022) [33]	Masson's staining showed larger fibrous tissue growth in P5 BMSC-EVs treated tendons compared to P12 BMSC-EVs and PBS treated tendons. Achilles tendon treated with P5 BMSC-EVs also had higher scores on tendon healing scoring system compared to P12 BMSC-EVs and PBS treated tendons. PBS and P12 BMSC-EVs groups had higher chondrification scores than P5 BMSC-EVs.	Early passaged MSC-EVs promoted better healing than late passaged/senescent MSC-EVs. This suggests that aging impairs the wound healing process and potentially even renders it useless.	Further studies of MSC-EV glycomes can give insight into the therapeutic benefits they provide.
Yao et al. (2021) [24]	hUMSC-Exos increased collagen deposition, promoted differentiation of TSCs into tendon, accelerated expression of tendon specific genes. hUMSC-Exos also promoted the growth of dense, regular collagen while the control groups grew loose, sparse collagen.	This study provides a potential explanation of the therapeutic benefit of stem cells in tendon healing.	-
Xu et al. (2023) [34]	Administration of EVB improved tenogenesis by reducing harmful morphological changes and preventing heterotopic ossification. EVB also improved biomechanical tests like load, stiffness, and tensile modulus compared to EVN.	Further studies should build upon this design of BG based priming of ATSC-EVs in regenerative medicine.	-
Chamberlain et al. (2019) [22]	Administration of extracellular vesicle-educated macrophages (EEMs) improved tendon function in ultimate stress and Young's Modulus compared to treatment with MSC-EVs, MQs, and control. However, MSC-EV treatment also induced a regenerative response	Macrophage administration has utility in tendon healing and other musculoskeletal injuries.	Further exploration of the macrophages modulated by MSCs is needed to better understand this process.

	seen through increased endothelial cells and a decreased M1/M2 ratio.		
Han et al. (2022) [35]	RhoA inhibition reduces cell proliferation and invasion, but treatment with hucMSC recovered cell proliferation in tendon injury of rats.	This study offers potential therapies to improve tendon healing.	
Shen et al. (2023) [23]	Administration of iEV promoted the expression of anti-inflammatory gene expression, reduced mononuclear cell accumulation at the injury, increased collagen deposition at the injury site, and decreased peritendinous scar formation in a dose dependent manner. iEV treatment also led to faster functional recovery with less incidence of re-rupture or tendon gap.	The study provides a basis for utility and modification of iEVs to fit needs in specific disease processes.	Future studies will include higher intensity running to test tendon healing and see if there is dose dependent response in this test.
Shi et al. (2020) [36]	BMSC-Exos administration decreased M1 macrophages and proinflammatory factors, but increased M2 macrophage numbers, anti-inflammatory factors, and chondrogenic factors compared to control groups. BMSC-Exos also showed increased collagen and aggrecan formation, decreased apoptosis, and increased cell proliferation compared to controls. Biomechanical testing showed improved maximum force, strength, and elastic modulus in BMSC-Exos compared to controls.	-	Further studies of the reason for M2 polarization of macrophages should be explored.
Li et al. (2020) [37]	HCPT-primed hUCSC-EVs improved anti-adhesion following	This is a new option for anti-adhesion in tendon injury healing.	-

	Achilles tendon injury when compared to control (unprimed EVs). HCPT-EVs inhibited myofibroblast differentiation as well as fibroblast proliferation and viability.		
Zhang et al. (2020) [38]	TSC-Exos increase proliferation and cell migration in a dose dependent manner. 1-week post-op, TSC-Exos decreased inflammation and apoptosis compared to control and GelMA. Histological analysis 2 weeks and 8 weeks post op showed that TSC-Exos promoted more continuous and regular tendon growth than GelMA and control. Analysis at 8 weeks showed a larger diameter of collagen in TSC-Exos treated tendon compared to control and GelMA.	Clinical application of TSC-Exos in tendon repair.	Multiple time points need to be established to monitor the phosphorylation of AKT and ERK. Other therapeutic pathways need to be examined. Direct biomechanical testing of healed testing needs to be performed. Characterization of exosomal proteins needs to be done.
Xu et al. (2022) [39]	Tendons treated with exosomes had better histological scores, lower fibril density, and larger collagen diameter compared to the ectosome group at 3 and 5 weeks after administration. 5 weeks after administration, there was more type 1 collagen in the exosome group compared to the ectosome group. Biomechanical testing showed higher failure load, tensile stress, and elastic modulus in the exosome-treated tendon compared to ectosome treatment.	Better the understanding of clinical treatment of tendinopathy with EVs.	Various EV dosages should be explored, including multiple injections at various time points.
Shen et al. (2020) [40]	ATSC-EVs primed with interferon gamma attenuated the immune response, increased collagen	Potential new treatment option for tendon injury.	Broad spectrum proteomics of tenocytes and macrophages after EV treatment to account

	formation at injury site, reduced rupture, and reduced tendon gap formation compared to control and non-primed ATSC-EVs.		for alternative mechanisms.
Liu et al. (2021) [41]	Stem cell-derived Exos modified by a nitric oxide nanomotor delivered by microneedles decreased inflammation in Achilles tendinopathy, increased Col1a expression, improved organization of fibers, and preserved the ECM.	Nanometers integrated with microneedles have many applications in enthesopathy healing and other biomedical fields.	-
Gissi et al. (2020) [42]	High doses of rBMSC-EVs accelerated tendon remodeling, improved fiber alignment, increased collagen type 1, and decreased collagen type 3 when compared to control as well as application of low doses and stem cells.	Multiple uses in tendon healing.	-
Rong et al. (2023) [43]	ENEVs promoted function restoration, matrix repair, and tendon morphology while suppressing scarring and peritendinous adhesion.	Application in tissue regeneration in multiple disease processes.	-
Chen et al. (2021) [9]	Administration of EVs from ATSC-EVs improved mechanical function, promoted tenocyte proliferation, and increased the expression of decorin, collagen I, tenomodulin, and biglycan.	-	Further evaluate the dose dependent response seen in this study. Add more time points to evaluate the healing process. Evaluate cargo of EVs to determine mechanisms.

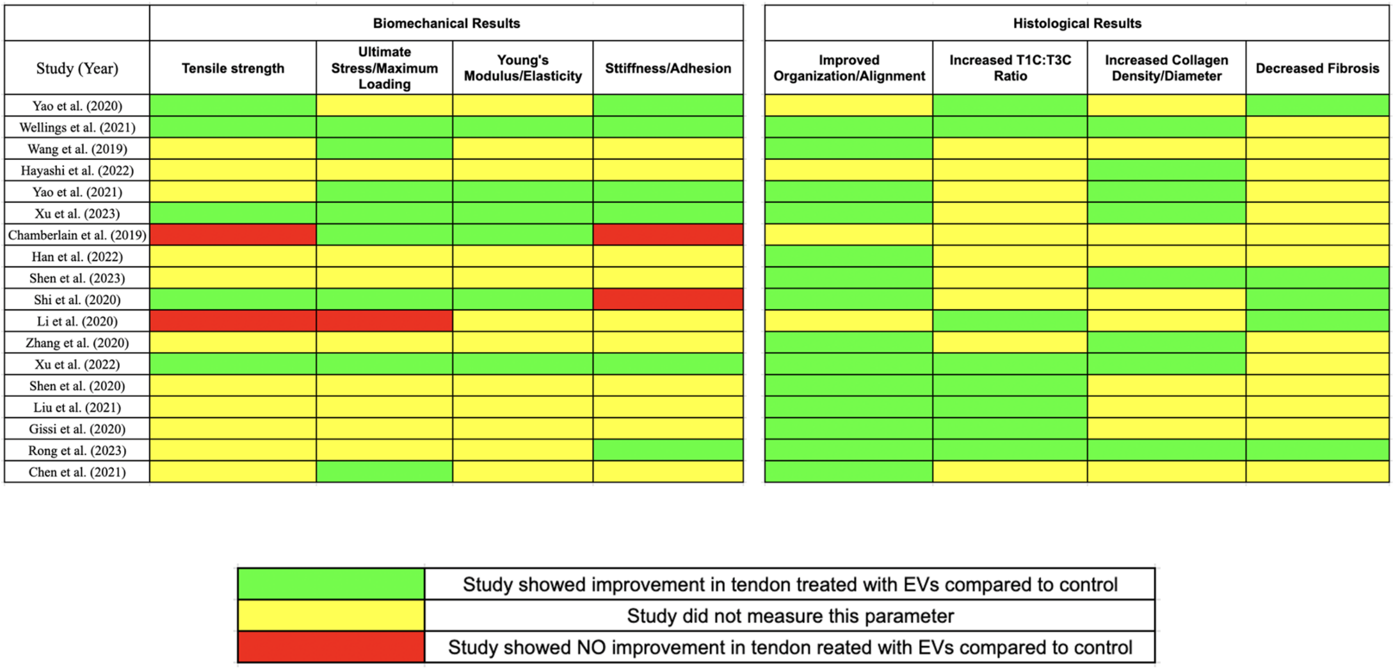

Supplementary Figure S1: Heatmap representation of specific biomechanical and histological results for included studies.

References

1. Ouzzani, M.; Hammady, H.; Fedorowicz, Z.; Elmagarmid, A. Rayyan—A web and mobile app for systematic reviews. *Syst. Rev*. **2016**, *5*, 210. https://doi.org/10.1186/s13643-016-0384-4.
2. Page MJ, McKenzie JE, Bossuyt PM, et al. The PRISMA 2020 statement: an updated guideline for reporting systematic reviews. *BMJ*. Published online March 29, 2021:n71. doi:10.1136/bmj.n71
3. Hooijmans, C.R.; Rovers, M.M.; Vries, R.B.; Leenaars, M.; Ritskes-Hoitinga, M.; Langendam, M.W. SYRCLE's risk of bias tool for animal studies. *BMC Med. Res. Methodol*. **2014**, *14*, 43. https://doi.org/10.1186/1471-2288-14-43.
